# Blink index as a response predictor of blepharospasm to botulinum neurotoxin‐A treatment

**DOI:** 10.1002/brb3.2374

**Published:** 2021-09-23

**Authors:** Jeongkyeong Jang, Helen Lew

**Affiliations:** ^1^ Department of Ophthalmology CHA Bundang Medical Center, College of Medicine, CHA University Seongnam Republic of Korea

**Keywords:** blepharospasm, blink index, blink profile, botulinum neurotoxin‐A

## Abstract

**Purpose:**

We investigated the blink profiles and blink index using ocular surface interferometer in the patients with blepharospasm (BSP) and identified points to consider predictive factor after BSP treatment.

**Methods:**

In total, 117 eyelids of 59 elderly patients and 20 eyelids of 10 age‐matched control group were studied. All BSP patients applied botulinum toxin‐A (BoNT‐A) injection for treatment of BSP. An ocular surface interferometer (LipiView; TearScience, Morrisville, NC, USA) was used to measure blink profile and blink index; total and incomplete blinks/20 s, and the partial blink ratio (PBR). Eyelid blink time (including lid closing time, closure time, lid opening time), interblink times (IBT), closing speeds (OS), and opening speeds (OS) were analyzed using 600 blinks recorded over 20 s.

**Results:**

Total blink rate was significantly higher in BSP patients compared to the age‐matched control group (*p* = .029) but other time‐related and speed‐related index including interpalpebral fissure, PBR, blink time, closure time (CT), interblink time, CS, and OS were not significantly different. In the responder of BSP patients, the average age was higher, CT was shorter, CS was faster than nonresponder (age; *p* = .016, CT; *p* < .001, CS; *p* = .042).

**Conclusion:**

The blink index by analyzing the blink profile using ocular surface interferometer, and this blink index may be used as a predictive factor for evaluating the clinical response after BoNT‐A injection in blepharospasm patients.

## Graphical abstract

This study provides us new understandings about the blink profile of blepharospasm patients using ocular surface interferometer. The blink index is a new predictive factor for the clinical response after botulinum ueurotoxin‐A treatment.

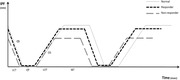

## 1 INTRODUCTION

Blepharospasm (BSP) is the focal dystonia involving periocular muscles and result in involuntary and abnormal eyelid movement. BSP patients have increased blink rate at rest and during conversation compared to healthy people (Bentivoglio et al., 2006). In addition, increased blink rate may precede BSP that the results of more than 5 years of follow‐up on the patients with increased blinking (Conte et al., 2017). Conte et al. (2013) reported that the blink reflex recovery cycle by stimulating the supraorbital nerve and analyzed using EMG response and suggested that the changes of R2 recovery cycle in the BSP patients due to contraction of the orbiculraris oculi.

Botulium neurotoxin‐A (BoNT‐A) injection is considered the primary treatment for essential blepharospasm and more than 90% of clinical improvements (Cillino et al., 2010). BoNT‐A injection reduces about 40% of blink rate and 60% of Blepharospasm Disability Index (BSDI) in all BSP and increased blinking (Ferrazzano et al., 2015) and Individualized BoNT‐A injection reduces clinical symptoms (Sung et al., 2015).

Apraxia of eyelid opening, as a variant feature of blepharospasm, carries a complex differential diagnosis that is most consistent with late‐onset dystonia and BoNT‐A injection is considered effective treatment (Forget et al., 2002). Pathogenesis of apraxia of eyelid opening and blepharospasm is poorly understood, but there a few hypotheses based on animal model studies. Nigro‐striatal basal ganglia pathways may project to the premotor control of eyelid coordination. Therefore, it is associated with dysfunction in the corticothalamic, basal ganglia, and focal cranial nerve circuitry (Weiss et al., 2010).

However, clinical symptoms do not improve in all BSP patients after BoNT injection, so we want to investigate the dynamics of blink in the BSP patients using ocular surface interferometer, analyze the blink profile, define the blink index, and evaluate the correlation between blink index and clinical responses of BoNT‐A treatment response.

## 2 MATERIALS AND METHODS

### 2.1 Patients and clinical evaluations

This retrospective medical chart review was performed from February 2017 to June 2020 at Bundang CHA Medical Center, Seongnam, Republic of Korea. This study adhered to all relevant tenets of the Declaration of Helsinki and was approved by our institutional review board. We included 117 eyelids of 59 patients with treated with BoNT‐A injection (OnabotulinumtoxinA, INNOTOX^®^ 25 units, Medytox Inc., Seoul, Korea). All subjects could be tested using a LipiView Interferometer (TearScience, Morrisville, NC, USA) before BoNT‐A injection in the outpatient clinic. In this retrospective medical chart review, the controls were age‐matched other patients who did not have any periocular muscle dystonia disease. Patients with dry eye syndrome, secondary dystonia, psychiatric conditions, and other condition of ocular disease, which can cause abnormal blinking of the periocular muscles, were excluded. Only patients followed up for at least 1 month after BoNT‐A injection were included. Clinical outcomes were evaluated by Jankovic rating scale (JanKovic et al., 1990) or change of injection dose. The frequency and severity score of all patients were higher than 3 points. We defined the responder group improved to 0 or 1 point in the rating scale and maintained the initial injection dose next time injection. Nonresponder group was defined as presenting higher than 2 points in the rating scale at initial injection and next injection with the 50% increased dose of BoNT‐A.

### 2.2 Blink dynamics analysis

**FIGURE 1:**
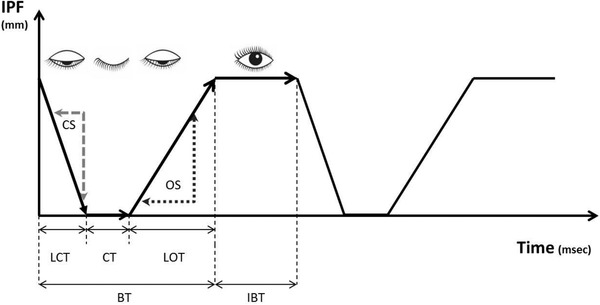
Blink profiles obtained using ocular surface interferometer. BT; blink time, IBT; interblink time, LCT; lid closing time, CT; closure time, LOT; lid opening time, CS; closing speed, OS; opening speed

An analysis of blink dynamics was based on the 20 s videos (600 frames), which recorded in LipiView interferometer. Total blink rate (TBR) and partial blink ratio (PBR) was counted in the internal program of LipiView interferometer. We set the blink cycle as the blink time (BT) and interblink time (IBT), which means keeping the eyelid open. BT was defined as consisting of lid closing time (LCT) taken by the interpalpebral fissure (IPF) to reach the maximum closure from the minimum closure, closure time (CT); the time for which the upper eyelid remained completely closed, and lid opening time (LOT); the time taken by the upper eyelid to change frome the minimum to maximum IPF. The dynamic index of closing and opening eyelid was defined as closing speed (CS) and opening speed (OS). CS and OS were calculated as IPF (mm) per LCT (s) and IPF (mm) per LOT (s). All measurements were performed on all blinks made within the 20 s, and averaged. Each blink profile is presented as an IPF versus time graph (Figure 1). A desktop computer running Windows 10 and the video software was used for data capture and analysis. All measurements were derived by a single examiner (JJ).

### 2.3 Statistical analysis

All statistical analyses employed SPSS for Windows, version 25.0 (IBM Corp., Armonk, NY, USA). The Mann–Whitney test was used to compare the control, responder, and nonresponder in BSP groups. A *p* value < .05 was regarded as statistically significant.

## 3 RESULTS

**FIGURE 2:**
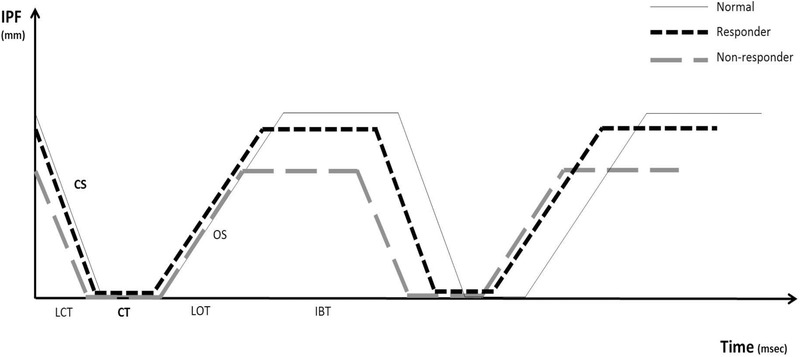
Comparison of blink profiles according to botulinum neurotoxin‐A (BoNT‐A) injection response. Blink rate was increased in blepharospasm patients. Increased closure time and decreased closing speed were noted in nonresponders compared with responders after BoNT‐A injection. LCT; lid closing time, CT; closure time, LOT; lid opening time, IBT; interblink time, CS; closing speed, OS; opening speed

A total 117 eyelids of 59 BSP patients (37 of male, 80 of female) who met the inclusion and exclusion criteria were enrolled this study. The average age of patients receiving BoNT‐A therapy was 65.3 ± 11.3 years. After BoNT‐A therapy, 82.9% of the patients were responders, but the others of 17.1% were nonresponders. Average injection dose of BoNT‐A was higher in nonresponder group (responder; 0.7 ± 0.2 cc, nonresponder; 0.9 ± 0.3 cc, *p* = .004) and number of injection was also higher in nonresponder (responder; 2.7 ± 2.1 times, nonresponder; 4.3 ± 3.9 times, *p* = .028) (Table 1). In the blink profile analyzed by LipiView^®^, TBR was significantly higher in BSP patients compared to the age‐matched control group (BSP; 10.4 ± 4.8 times per 20 s, control; 7.4 ± 3.6 times per 20 s, *p* = .029) but other time‐related and speed‐related index including IPF, PBR, BT, CT, IBT, CS, and OS were not significantly different. In the BSP patients, the average age of responder was higher, CT was shorter, CS was faster than nonresponder (age; *p* = .016, CT; *p* < .001, CS; *p* = .042). On the other hand, there was no significant difference in the other blink profiles compared to responder and nonresponder (Table 2, Figure 2). Normalized TBR (6.8 ± 4.4 times/20 s) was observed 1 month after the BoNT‐A injection in responder, but other indexes were not changed significantly (Table 3).

**TABLE 1:** Clinical characteristics in the patients with blepharospasm according to botulinum neurotoxin‐A treatment

		Blepharospasm				
	Control	Responder	Nonresponder	Total	*p* * Value	*p* ** Value
N (eyes)	20	97	20	117		
Sex (M:F)	6:14	27:70	10:10	37:80	*.081*	.082
Age (yr)	63.5 ± 9.4	61.8 ± 11.6	69.5 ± 9.5	65.3 ± 11.3	***.016***	.652
IPF (mm)	7.2 ± 1.6	7.3 ± 1.7	6.7 ± 1.4	7.0 ± 1.6	*.149*	.645
Vascular disease						
With (%)		13 (11.1)	2 (1.7)	15 (12.8)	.348	
Without (%)		73 (62.4)	29 (24.8)	102 (87.2)		
Average injection dose of BoNT‐A (cc)		0.7 ± 0.2	0.9 ± 0.3	0.7 ± 0.2	**.004**	
Numbers of treatment		2.7 ± 2.1	4.3 ± 3.9	3.0 ± 2.6	**.028**	

IPF, interpalpebral fissure; BoNT‐A, Botulium Toxin‐A.

* Mann–Whitney test; responder versus nonresponder in blepharospasm.

** Mann–Whitney test; control versus blepharospasm.

The Bold and italic numbers are < 0.05.

**TABLE 2:** Blink index in the patients with blepharospasm according to botulinum neurotoxin‐A treatment

		Blepharospasm				
	Control	Responder	Nonresponder	Total	*p* * Value	*p* ** Value
TBR (/20s)	7.4 ± 3.6	9.9 ± 5.1	10.9 ± 4.5	10.4 ± 4.8	*.238*	**.029**
PBR (%)	43.9 ± 33.7	44.5 ± 37.4	26.9 ± 26.7	36.6 ± 33.8	*.117*	.975
BT (ms)	656.4 ± 150.4	635.7 ± 155.6	635.6 ± 130.5	635.7 ± 143.1	*.850*	.606
CT (ms)	108.9 ± 17.8	65.6 ± 120.5	120.5 ± 49.6	106.9 ± 95.0	***<.001***	.075
IBT (ms)	2261.9 ± 2054.1	2864.9 ± 2375.9	2103.9 ± 910.4	2520.7 ± 1883.5	*.176*	.085
CS (mm/s)	68.8 ± 21.6	63.7 ± 15.1	55.1 ± 19.5	59.9 ± 17.6	***.042***	.558
OS (mm/s)	17.6 ± 5.0	18.3 ± 5.2	18.6 ± 6.4	18.5 ± 5.7	*.771*	.474

TBR, total blink rate; PBR, partial blink ratio; BT, blink time; CT, closure time; IBT, interblink time; CS, closing speed; OS, opening speed.

* Mann–Whitney test; responder versus nonresponder in blepharospasm.

** Mann–Whitney test; control versus blepharospasm.

The Bold and italic numbers are < 0.05.

**TABLE 3:** Comparison of blink indexes before botulinum neurotoxin‐A (BoNT) treatment and after 1 month

	Responder (*n* = 38 eyes)			Nonresponder (*n* = 6 eyes)		
	Before BoNT injection	After 1 month	*p* * Value	Before BoNT injection	After 1 month	*p* Value
TBR (/20s)	10.8 ± 6.5	6.8 ± 4.4	**.003**	9.7 ± 1.5	8.2 ± 4.3	.394
PBR (%)	49.7 ± 32.9	50.7 ± 38.9	.852	44.9 ± 29.7	33.4 ± 36.7	.394
BT (ms)	688.8 ± 251.1	595.2 ± 154.9	.326	833.3 ± 324.9	701.8 ± 190.8	.833
CT (ms)	142.8 ± 180.0	103.2 ± 95.9	.789	211.1 ± 194.9	183.3 ± 139.4	.917
IBT (ms)	2492.9 ± 2230.9	4209.9 ± 4802.3	.101	2094.3 ± 382.9	3041.2 ± 1465.6	.173
CS (mm/s)	62.9 ± 19.0	62.7 ± 21.3	.917	44.0 ± 21.8	46.9 ± 7.8	.345
OS (mm/s)	19.8 ± 5.8	22.6 ± 7.7	.196	14.3 ± 4.6	16.9 ± 6.1	.249

TBR, total blink rate; PBR, partial blink ratio; BT, blink time; CT, closure time; IBT, interblink time; CS, closing speed; OS, opening speed.

* Wilcoxon signed‐rank test; analyzing the blink indexes of same patients who were able to take the LipiView interferometer test one month after BoNT‐A injection.

The Bold and italic numbers are < 0.05.

## 4 DISCUSSION

The purpose of present study is to analyze predisposing and treatment prognostic factors of patients with BSP using ocular surface interferometer, which can be easily performed in an outpatient clinic. BSP patients in this study had a tendency to increased total blink rate compared to age‐matched control group as previous study (Conte et al., 2013). However, other time‐ and speed‐related indexes of the ocular surface parameters were not different from those the control group.

Conte et al. reported that the blink reflex recovery cycle using EMG response and suggested increased R2 recovery cycle in the patients with BSP. The R2 recovery index and blink rate was correlated, suggesting that the changes of R2 recovery cycle in the BSP patients due to contraction of the orbicularis oculi (Conte et al., 2013). Just as previous EMG studies have helped us understand BSP pathogenesis, it suggest that analyze the blink dynamics also helps us understand BSP pathogenesis. Although the previous study did not show a response of BSP treatment, this study analyzed not only blink dynamics of BSP but also the treatment response. Comparing the responder and nonresponder groups after BoNT‐A injection, it was confirmed that the increased CT and decreased CS in the nonresponder groups. It seems that shorter CT and longer IBT in the responder groups may appear to be related to an increased blink rate, which is irregular, and orbicularis dystonia in general clinical features of spasm compared to the control group. But on the contrary, longer CT, shorter IBT, and shorter CS in nonresponders may explain that the levator function is also affected.

After 1 month treated with BoNT‐A injection, normalized TBR was observed in in responder groups, but other indexes were not changed in both treated groups. CT was also tended close to the control group in responder groups but statistically not significant. It indicates the blink pattern does not change after BoNT‐A treatment except TBR. This suggests that the blink character in BSP patients is unique compared to the control group, and that the etiology of muscle dystonia between responders and nonresponders may not be same. From these results, it can be considered that the etiology of BSP is not only dystonia of orbicularis oculi contraction but the decreased function of the eyelid levator muscles in the nonresponder group. As the results in the nonresponder group, we suggest that the CT and CS analyzed blink index can be used as prognostic factors for BoNT‐A injection treatment.

The other finding between responder and nonresponder groups was age, and it was found that the nonresponders was slightly older. This may be interpreted as changes in neurotransmitter (NT) production, NT transmission, and NT receptor functions as age increases. In previous animal model, having both depleting the dopamine NT and eye irritation condition produce the BSP, and cerebellum blocked blink amplitude and duration (Evinger, 2013; Hall et al., 2006). It can be interpreted that NT depletion according to age can contribute to the etiology of BSP.

According to the results of this study, CT, CS, and age can be used as prognostic factors for the treatment of BoNT‐A injection and can be helpful in etiology interpretation of BSP patients. Considering the relationship between age and blink, there may be an effect of NT which may be related to genetic change. As neurologic diseases such as apraxia of eyelid opening in elderly patients are increased, BSP can be also related to NT and further studies about the relationship between systemic disease and BSP are considered to be necessary. A study to confirm the genetic factors of hemifacial spasm patients reported that there was no significant difference from healthy people (Han et al., 2008), whereas other study reported that gene mutation of Adenylate cyclase 5 (ADCY5) in patients with familial dyskinesia with facial myocardia (Chen et al., 2012). Therefore, genetic analysis in BSP patients can also be used as a prognostic factor and remains a question for future study.

There are limitations to this study. It was a retrospective study, the follow‐up duration is not the same in all patients, so changes in blink dynamics before and after BoNT‐A treatment are not definitely identified. Because not all patients could come to follow‐up and test interferometer until the next BoNT‐A injection on account of medical insurance issues, we could analyze the blink indexes of patients who were able to take the LipiView interferometer test one month after BoNT‐A injection. The other limitation is the injection dose of BoNT‐A was variable depending on the degree and extent of BSP. We should determine the long‐term results and identify possible correlation factor of treatment response of BSP patients with blink dynamics in future studies.

Despite these limitations, this study suggest that the blink dynamics can be used for analyzing etiology and treatment response of BSP.

## 5 CONCLUSIONS

In conclusion, we found the blink index by analyzing the blink profile using ocular surface interferometer which can be easily tested in clinic, and this blink index may be used as a predictive factor for evaluating the clinical response after BoNT‐A injection in BSP patients.

## CONFLICT OF INTEREST

The authors declare that they have no conflict of interest.

### PEER REVIEW

The peer review history for this article is available at https://publons.com/publon/10.1002/brb3.2374.
